# What do Cochrane systematic reviews say about new practices on integrative medicine?

**DOI:** 10.1590/1516-3180.2018.0172170418

**Published:** 2018-06-25

**Authors:** Rachel Riera, Vinícius Lopes Braga, Luana Pompeu dos Santos Rocha, Daniel Damasceno Bernardo, Luísa Avelar Fernandes de Andrade, Jessica Chiu Hsu, Luciana Di Giovanni Marques da Silva, Rodrigo Cesar de Sá Suetsugu, Nicole Hosni Dittrich, Lucas Riguete Pereira de Lima, Vicente Penido da Silveira, Barbara Caon Kruglensky, Letícia de Freitas Leonel, Edivando de Moura Barros, Anderson Adriano Leal Freitas da Costa, Miguel Lins Quintella, Rafael Leite Pacheco, Carolina de Oliveira Cruz, Ana Luiza Cabrera Martimbianco, Daniela Vianna Pachito, Vania Mozetic, Tatiana de Bruyn Ferraz Teixeira, Maria Regina Torloni, Alvaro Nagib Atallah

**Affiliations:** I MD, MSc, PhD. Rheumatologist; Adjunct Professor, Discipline of Evidence-Based Medicine, Escola Paulista de Medicina (EPM), Universidade Federal de São Paulo (Unifesp); and Researcher, Cochrane Brazil, São Paulo (SP), Brazil.; II Undergraduate Medical Student, Escola Paulista de Medicina (EPM), Universidade Federal de São Paulo (Unifesp), São Paulo (SP), Brazil.; III Undergraduate Medical Student, Escola Paulista de Medicina (EPM), Universidade Federal de São Paulo (Unifesp), São Paulo (SP), Brazil.; IV Undergraduate Medical Student, Escola Paulista de Medicina (EPM), Universidade Federal de São Paulo (Unifesp), São Paulo (SP), Brazil.; V Undergraduate Medical Student, Escola Paulista de Medicina (EPM), Universidade Federal de São Paulo (Unifesp), São Paulo (SP), Brazil.; VI Undergraduate Medical Student, Escola Paulista de Medicina (EPM), Universidade Federal de São Paulo (Unifesp), São Paulo (SP), Brazil.; VII Undergraduate Medical Student, Escola Paulista de Medicina (EPM), Universidade Federal de São Paulo (Unifesp), São Paulo (SP), Brazil.; VIII Undergraduate Medical Student, Escola Paulista de Medicina (EPM), Universidade Federal de São Paulo (Unifesp), São Paulo (SP), Brazil.; IX Undergraduate Medical Student, Escola Paulista de Medicina (EPM), Universidade Federal de São Paulo (Unifesp), São Paulo (SP), Brazil.; X Undergraduate Medical Student, Escola Paulista de Medicina (EPM), Universidade Federal de São Paulo (Unifesp), São Paulo (SP), Brazil.; XI Undergraduate Medical Student, Escola Paulista de Medicina (EPM), Universidade Federal de São Paulo (Unifesp), São Paulo (SP), Brazil.; XII Undergraduate Medical Student, Escola Paulista de Medicina (EPM), Universidade Federal de São Paulo (Unifesp), São Paulo (SP), Brazil.; XIII Undergraduate Medical Student, Escola Paulista de Medicina (EPM), Universidade Federal de São Paulo (Unifesp), São Paulo (SP), Brazil.; XIV Undergraduate Medical Student, Escola Paulista de Medicina (EPM), Universidade Federal de São Paulo (Unifesp), São Paulo (SP), Brazil.; XV Undergraduate Medical Student, Escola Paulista de Medicina (EPM), Universidade Federal de São Paulo (Unifesp), São Paulo (SP), Brazil.; XVI Undergraduate Medical Student, Escola Paulista de Medicina (EPM), Universidade Federal de São Paulo (Unifesp), São Paulo (SP), Brazil.; XVII MD. Postgraduate Student, Evidence-Based Health Program, Universidade Federal de São Paulo (Unifesp), and Assistant Researcher, Cochrane Brazil, São Paulo (SP), Brazil.; XVIII MSc. Psychologist; Postgraduate Student, Evidence-Based Health Program, Universidade Federal de São Paulo (Unifesp); and Assistant Researcher, Cochrane Brazil, São Paulo (SP), Brazil.; XIX MSc, PhD. Physiotherapist; Postdoctoral Student, Evidence-Based Health Program, Universidade Federal de São Paulo (Unifesp); and Volunteer Researcher, Cochrane Brazil, São Paulo (SP), Brazil.; XX MD, MSc. Neurologist; Postgraduate Student, Evidence-Based Health Program, Universidade Federal de São Paulo (Unifesp); and Assistant Researcher, Cochrane Brazil, São Paulo (SP), Brazil.; XXI MD. Ophthalmologist; Postgraduate Student, Evidence-Based Health Program, Universidade Federal de São Paulo (Unifesp); and Volunteer, Cochrane Brazil, São Paulo (SP), Brazil.; XXII Journalist; Professor, Fundação Casper Libero, São Paulo (SP); Postgraduate Student, Evidence-Based Health Program, Universidade Federal de São Paulo (Unifesp); and Volunteer, Cochrane Brazil, São Paulo (SP), Brazil.; XXIII MD, MSc, PhD. Obstetrician; Affiliated Professor, Discipline of Evidence-Based Medicine, Escola Paulista de Medicina (EPM), Universidade Federal de São Paulo (Unifesp); and Researcher, Cochrane Brazil, São Paulo (SP), Brazil.; XXIV MD, MSc, PhD. Nephrologist; Full Professor, Discipline of Evidence-Based Medicine, Escola Paulista de Medicina (EPM), Universidade Federal de São Paulo (Unifesp); and Director, Cochrane Brazil, São Paulo (SP), Brazil.

**Keywords:** Review [publication type], Public health administration, Evidence-based medicine, Integrative medicine, Health policy

## Abstract

**BACKGROUND:**

This study identified and summarized all Cochrane systematic reviews (SRs) on the effects of ten integrative practices that were recently added to the Brazilian public healthcare system (SUS).

**DESIGN AND SETTING:**

Review of systematic reviews, conducted in the Discipline of Evidence-Based Medicine, Escola Paulista de Medicina (EPM), Universidade Federal de São Paulo (Unifesp).

**METHODS:**

Review of Cochrane SRs on the following interventions were identified, summarized and critically assessed: apitherapy, aromatherapy, bioenergetics, family constellation, flower therapy, chromotherapy, geotherapy, hypnotherapy, hand imposition or ozone therapy.

**RESULTS:**

We included a total of 16 SRs: 4 on apitherapy, 4 on aromatherapy, 6 on hypnotherapy and 2 on ozone therapy. No Cochrane SR was found regarding bioenergetics, family constellation, chromotherapy, clay therapy, flower therapy or hand imposition. The only high-quality evidence was in relation to the potential benefit of apitherapy, specifically regarding some benefits from honey dressings for partial healing of burn wounds, for reduction of coughing among children with acute coughs and for preventing allergic reactions to insect stings.

**CONCLUSION:**

Except for some specific uses of apitherapy (honey for burn wounds and for acute coughs and bee venom for allergic reactions to insect stings), the use of ten integrative practices that have recently been incorporated into SUS does not seem to be supported by evidence from Cochrane SRs.

## INTRODUCTION

On March 2018, the Brazilian Ministry of Health announced an expansion of its policies for integrative practices for healthcare within the Brazilian public healthcare system (Sistema Único de Saúde, SUS). Thus, ten new types of integrative practices now form part of the list of procedures available through SUS: apitherapy, aromatherapy, bioenergetics, family constellation, chromotherapy, clay therapy, hypnotherapy, hand imposition, ozone therapy and flower therapy.^1^

The term “integrative practice” commonly refers to incorporation of complementary approaches into a healthcare system.^2^ It is important to differentiate between the concepts of “alternative” and “complementary” practices. When a non-mainstream practice is used together with conventional medicine, it is considered to be “complementary.” Conversely, when a non-mainstream practice is used in place of conventional medicine, it is considered to be “alternative.” Purely alternative approaches are seen less frequently, given that most people using non-mainstream approaches do so alongside conventional approaches.^2^

Most complementary healthcare practices can be classified as use of natural products or as use of mind and body practices. They may include use of probiotics, dietary supplements, yoga, chiropractic and osteopathic manipulation, meditation, massage therapy, acupuncture, healing touch, hypnotherapy, etc.^2^

Use of integrative practices may be justified for patients with chronic non-transmissible conditions whose clinical manifestations remain resistant or unresponsive to conventional treatments. However, their effectiveness and safety, and subsequently their cost-effectiveness and budgetary impact, need to be evaluated in order to guide their incorporation into public or private healthcare systems.

In this review, we identified and summarized all the Cochrane systematic reviews on the benefits and harm from use of ten integrative approaches that have recently been made available for users of SUS.

## OBJECTIVE

To summarize the evidence from Cochrane systematic reviews focusing on ten integrative practices for preventive or therapeutic purposes, for any disease or condition.

## METHODS

### Design

Review of Cochrane systematic reviews.

### Setting

Discipline of Evidence-Based Medicine of Escola Paulista de Medicina (EPM), Universidade Federal de São Paulo (UNIFESP), and Cochrane Brazil.

### Criteria for including reviews

#### Types of studies

We considered the latest version of full Cochrane systematic reviews (SR). We excluded any protocol or any SR marked as “withdrawn” in the Cochrane Database of Systematic Reviews (CDSR).

#### Types of participants

We considered any healthy participant who received integrative practices for preventive purposes and any participant presenting any condition of illness who received integrative practices for therapeutic purposes.

#### Types of interventions

We included the integrative practices listed below that were used for preventive or therapeutic purposes and compared their use with no intervention or use of placebo or any other pharmacological or non-pharmacological intervention that was considered to represent a conventional, alternative or complementary approach. The integrative practices considered in this review are the same that are now provided in the Brazilian public health system, and comprised: apitherapy, aromatherapy, bioenergetics, family constellation, chromotherapy, clay therapy, hypnotherapy, hand imposition, ozone therapy and floral therapy. We only considered reviews that exclusively focused on one of these integrative interventions, rather than those addressing multiple interventions (called “umbrella” reviews).

#### Type of outcomes

We considered any clinical, social, laboratory or economic outcomes, as evaluated in the systematic reviews that were included.

### Search for reviews

We carried out a sensitive systematic search in the Cochrane Database of Systematic Reviews (via Wiley) on March 14, 2018. The search strategy is presented in Table 1.

Additionally, we conducted a manual search among titles listed on the web page “Cochrane Reviews and Protocols related to Complementary Medicine”, which is available from the Cochrane Complementary Medicine website: http://cam.cochrane.org/cochrane-reviews-and-protocols-related-complementary-medicine.

**Table 1:** Search strategy

#1 MeSH descriptor: [Apitherapy] explode all trees
#2 MeSH descriptor: [Aromatherapy] explode all trees
#3 MeSH descriptor: [Color Therapy] explode all trees
#4 MeSH descriptor: [Therapeutic Touch] explode all trees
#5 MeSH descriptor: [Flower Essences] explode all trees
#6 (Apitherapy) OR (Apitoxins) OR (Apipuncture) OR (Bee Venom Therapy) OR (Bee Venom) OR (Honey) OR (Propolis) OR (Aromatherapy) OR (Bioenergetic) OR (Bioenergetic Therapy) OR (Bioenergetic Analysis) OR (Bioenergetic Psychotherapy) OR (Family Constellation) OR (Family Constellation Therapy) OR (Therapy, Color) OR (Chromatotherapy) OR (Chromotherapy) OR (Colour Light Therapy) OR (Geotherapy) OR (Hypnotherapy) OR (Hypnosis) OR (Healing Touch) OR (Hand Imposition) OR (Energy Channel) OR (Therapeutic Touch) OR (Energy Heal) OR (Laying-on-of-Hands) OR (Touch, Therapeutic) OR (Ozone) OR (Ozone Therapy) OR (Flower Essences) OR (Essences, Flower) OR (Bach Flower Remedies) OR (Flower Remedies, Bach) OR (Remedies, Bach Flower) OR (Bach Flowers) OR (Flowers, Bach) OR (Bach Flower Essences) OR (Essences, Bach Flower) OR (Flower Essences, Bach) OR (Flowering Top) OR (Top, Flowering) OR (Tops, Flowering) OR (Magnoliopsida) OR (Flowering Plants) OR (Flowering Plant) OR (Plant, Flowering) OR (Plants, Flowering) OR (Rosaceae) OR (Quince, Flowering) OR (Flowering Quince) OR (Flowering Quinces) OR (Quinces, Flowering) OR (Passiflora) OR (Passion Flower) OR (Flower, Passion) OR (Flowers, Passion) OR (Passion Flowers) OR (Platycodon) OR (Balloon Flower) OR (Balloon) OR (Flower, Balloon) OR (Flowers, Balloon) OR (Fraxinus) OR (Flowering Ash) OR (Ash, Flowering) OR (Ashs, Flowering) OR (Flowering Ashs) OR (Inflorescence) OR (Flower Head) OR (Flower Heads) OR (Head, Flower) OR (Heads, Flower) OR (Florigen) OR (Flowering Hormone) OR (Hormone, Flowering) OR (Integrative)
#7 #1 or #2 or #3 or #4 or #5 or #6
Filters: in Cochrane Reviews; *in Title, Abstract, Keywords*

### Selection of systematic reviews

Two researchers (RLP and COC) independently screened and evaluated all records retrieved through the systematic search, to confirm their eligibility in accordance with the inclusion criteria. Any disagreements were resolved by consulting a third author (RR or DVP).

### Presentation of the results

We presented a summary of the reviews included, through a narrative approach (qualitative synthesis). The key points considered were the respective PICOs (population, intervention, comparator and outcomes), methods for SR and meta-analyses, quality of primary studies included, quality of the body of the evidence for each outcome, and applicability. When multiple interventions were addressed by a single SR, we considered only those relevant for the present study.

## RESULTS

### Search results

The initial search retrieved 189 reviews and 13 protocols. After eliminating the protocols and assessing the full texts of the reviews, we excluded 173 reviews that did not fulfill our inclusion criteria. Thus, 16 Cochrane systematic reviews^3^^,^^4^^,^^5^^,^^6^^,^^7^^,^^8^^,^^9^^,^^10^^,^^11^^,^^12^^,^^13^^,^^14^^,^^15^^,^^16^^,^^17^^,^^18^ were included and summarized, as follows.

### Results from systematic reviews

The 16 systematic reviews included related to four integrative practices: apitherapy (four SRs), aromatherapy (four SRs), hypnotherapy (six SRs) and ozone therapy (two SRs). In relation to the other six integrative practices (bioenergetics, family constellation, chromotherapy, clay therapy, hand imposition and flower therapy), no SR was retrieved through the search strategy and, therefore, no conclusion can be presented regarding their efficacy or safety.

The main findings from the SRs included and the quality of the evidence (based on the GRADE approach) are presented in Table 2. A brief summary of each SR is presented below.

**Table 2:** Characteristics of interventions, comparisons, outcomes and quality of evidence

Integrative practice	Population and aim	Comparison	Benefits and harms	Evidence quality (GRADE approach)*
Honey (apitherapy)^3^	People with acute and/or chronic wounds	Conventional dressings for treatment of burns	• Honey dressings heal partial thickness burns more quickly than conventional dressings	High
			• No difference in overall risk of healing within six weeks for honey, compared with silver sulfadiazine	High
			• Burns treated with honey heal more quickly than those treated with silver sulfadiazine	Very low
			• Burns treated with honey presented lower risk of adverse events than the silver sulfadiazine group	High
Honey (apitherapy)^4^	Acute cough in children	Dextromethorphan, diphenhydramine, no treatment and placebo	• Use of honey was associated with reduced frequency of coughing, compared with the no treatment group	Moderate
			• Use of honey was associated with reduced frequency of coughing, compared with placebo	High
			• There was no difference between use of honey and use of dextromethorphan	Moderate
			• Use of honey was associated with reduced frequency of coughing, compared with diphenhydramine	Low quality
Venom immunotherapy (apitherapy)^5^	Preventing allergic reactions to insect stings	No intervention	• Use of venom immunotherapy versus no intervention reduced the risk of any systematic reaction to an insect sting	High
			• Reduction in the risk of large local reaction favoring venom immunotherapy	Moderate
			• The relative risk of any systematic reaction to treatment was higher with venom immunotherapy	Moderate
Aromatherapy^7^	Postoperative nausea and vomiting	Placebo, peppermint aromatherapy and isopropyl alcohol aromatherapy	• Aromatherapy reduced the use of rescue antiemetic medication, compared with placebo	Low
			• No difference between aromatherapy and placebo regarding:	
			a) Severity of nausea	Low
			b) Duration of nausea	Very low
			• No difference between peppermint aromatherapy and placebo regarding severity of nausea at five minutes	Low
			• Isopropyl alcohol aromatherapy showed benefits in relation to placebo for the following outcomes:	
			a) time (in minutes) to 50% reduction of nausea score	Moderate
			b) proportion of patients requiring antiemetics	Moderate
Aromatherapy^8^	Dementia	Placebo aromatherapy	This review included two RCTs with divergent results. No meta-analysis was performed because of heterogeneity and lack of data	Not assessed
Aromatherapy^10^	Pain management in labor	Standard care	No difference between groups regarding:	Not assessed
			a) assisted vaginal delivery risk	
			b) cesarean section risk	
			c) risk of neonatal intensive care admission	
Hypnosis (hypnotherapy)^13^	Pain management during labor and childbirth	Placebo, no treatment or any analgesic drug or technique	• Use of pharmacological pain relief or anesthesia was lower in the group that received self-hypnosis or hypnotherapy, compared with standard care	Very low
			• No difference was found between the groups regarding:	
			a) satisfaction with pain relief	Low
			b) spontaneous vaginal birth	Low
Hypnosis (hypnotherapy)^15^	Schizophrenia	Any treatment or standard therapy	• No difference was found between hypnosis and standard care in relation to the brief psychiatric rating scale	Not assessed
Hypnosis (hypnotherapy)^16^	Smoking cessation	No intervention and other intervention strategies	• Benefit in hypnotherapy group regarding the probability of smoking cessation at 12 months, compared with no treatment	Not assessed
			• Compared with psychological treatments, hypnotherapy alone did not improve smoking cessation at six months	
Ozone therapy^17^	Foot ulcers in people with diabetes	Antibiotic treatment or standard care	Compared with standard care, the ozone therapy group showed no difference regarding:	Not assessed
			• Ulcer area	
			• Number of ulcers healed	
			• Amputation rate	
			• Adverse events	

In this table, we only presented the results of systematic reviews that included studies that provided useful data. Thus, systematic reviews with no studies or with studies not containing any usable data were not included in this table. *GRADE (Grading of Recommendations Assessment, Development and Evaluation) has the aim of assessing the quality of the body of evidence. The evidence regarding a given outcome is assessed as having high quality (very high confidence in the results, i.e. the estimated effect is close to the true effect); moderate quality (it is very likely that the estimated effect is close to the real effect, but there is a possibility that it is not); low quality (the confidence in the effect estimate is limited); or very low quality (the true effect is likely to be substantially different from the estimate effect).

### 1. Apitherapy

Apitherapy refers to the use of byproducts from bees (honey, propolis or apitoxins) to promote health or as a treatment option for diseases.^19^ It is a broad term, including different practices ranging from topical use of honey as a wound treatment to systemic use of processed apitoxins for immunomodulation.

#### 1.1 Honey as a topical treatment for wounds

This review^3^ assessed the effects of honey, compared with alternative wound dressings and topical treatments, on the healing of acute and/or chronic wounds. It included 26 randomized clinical trials (RCTs, n = 3,011 participants). The RCTs included evaluated the effects of honey on:

- minor acute wounds (3 RCTs);
- burns (11 RCTs);
- different chronic wounds including venous leg ulcers (10 RCTs);
- diabetic foot ulcers (2 RCTs);
- infected postoperative wounds, pressure injuries, cutaneous leishmaniasis and Fournier’s gangrene (1 RCT each); and
- mixed populations of patients with acute and chronic wounds (2 RCTs).

The main findings were:

- honey dressings heal partial-thickness burns more quickly than conventional dressings (weighted mean difference [WMD] -4.68 days; 95% confidence interval [95% CI] -5.09 to -4.28; two RCTs; 992 participants; high quality of evidence).
- no difference in overall probability of healing within six weeks for honey, compared with silver sulfadiazine (relative risk [RR] 1.00; 95% CI 0.98 to 1.02; six RCTs; 462 participants; high quality of evidence).
- burns treated with honey heal more quickly than those treated with silver sulfadiazine (WMD -5.12 days; 95% CI -9.51 to -0.73; four RCTs; 332 participants; very low quality of evidence).
- burns treated with honey presented lower risk of adverse events than the silver sulfadiazine group (RR 0.29; 95% CI 0.20 to 0.42; six RCTs; 412 participants; high quality of evidence).

All other evidence was sparse, and its quality was downgraded because of risk of bias and imprecision. There was high diversity regarding participant inclusions and comparators within the RCTs included. The high-quality evidence available from comparison of honey versus silver sulfadiazine needs to be interpreted with caution, since this was a head-to-head comparison and no inactive group was considered. Until further studies with strong methodological quality are available, no robust conclusions for practice can be reached regarding other interventions or regarding wounds other than burns. For further details and to access all analyses, refer to the original abstract, available from: http://onlinelibrary.wiley.com/doi/10.1002/14651858.CD005083.pub4/full.

#### 1.2. Honey for treating acute coughing in children

This review^4^ had the aim of evaluating the effects of honey on acute coughing in children. Three RCTs were included, comparing honey with dextromethorphan, diphenhydramine, no treatment and placebo.

All the RCTs provided data for the primary outcome of symptomatic relief of frequency of coughing. A seven-point Likert scale was used (the lower the score was, the less severe the cough symptom under assessment was). The main results were:

- Use of honey was associated with reduced frequency of coughing, in comparison with the no treatment group (mean difference [MD] -1.05; 95% CI -1.48 to -0.62; two RCTs; 154 participants; moderate quality of evidence).
- Use of honey was associated with reduced frequency of coughing, in comparison with placebo (MD -1.85; 95% CI -3.36 to -0.33; one RCT; 300 participants; high quality of evidence).
- There was no difference between use of honey and use of dextromethorphan (MD -0.07; 95% CI -1.07 to 0.94; two RCTs; 149 participants; moderate quality of evidence).
- Use of honey was associated with reduced frequency of coughing in comparison with diphenhydramine (MD -0.57; 95% CI -0.90 to -0.24; one RCT; 80 participants; low quality of evidence).

Although some results indicate that use of honey may be associated with better results than those obtained through no treatment, placebo or diphenhydramine, caution should be used until solid recommendations for practice can be issued. All the available evidence was based on small RCTs, and the follow-up of some RCTs was only for one night after the intervention. It needs to be borne in mind also that the primary outcome was based on a scale: the minimum clinically relevant difference needs to be investigated and considered when recommending honey for symptomatic relief of coughing. Although some RCTs presented data regarding adverse events, no significant difference between the groups was reported. For further details and to access all analyses, refer to the original abstract, available from: http://cochranelibrary-wiley.com/doi/10.1002/14651858.CD007094.pub4/full.

#### 1.3. Venom immunotherapy for preventing allergic reactions to insect stings

This review^5^ evaluated the effects of venom immunotherapy (VIT) for preventing allergic reactions to insect stings. Six RCTs and one quasi-randomized controlled trial (n = 392 participants) were included. Use of VIT against no intervention reduced the risk of any systematic reaction to an insect sting (RR 0.10; 95% CI 0.03 to 0.28; seven RCTs; 206 participants; high quality of evidence). There was also a reduction in the risk of large local reaction, favoring VIT (RR 0.41; 95% CI 0.24 to 0.69; five RCTs; 112 participants; moderate quality of evidence). Regarding safety outcomes, systematic reaction to treatment was evaluated. The relative risk was higher in the VIT group (RR 8.16; 95% CI 1.53 to 43.46; six RCTs; 285 participants; moderate quality of evidence).

The authors of this review concluded that there was evidence supporting the use of VIT for preventing allergic reactions to insect stings. However, they considered that the low number of events in the groups would need to be taken into account and that further studies would be needed to reduce the imprecision in some results. For further details and to access all analyses, refer to the original abstract, available from: http://cochranelibrary-wiley.com/doi/10.1002/14651858.CD008838.pub2/full.

#### 1.4 Honey and lozenges for children with nonspecific coughs

This review^6^ aimed to evaluate the effects of honey and lozenges among children with chronic nonspecific coughs. The authors of this review conducted their search in 2009 and their strategy did not find any RCTs that fulfilled the inclusion criteria. Until further studies have been developed and this review has been updated, no solid conclusions for practice can be reached. For further information, refer to: http://onlinelibrary.wiley.com/doi/10.1002/14651858.CD007523.pub2/full.

### 2. Aromatherapy

Aromatherapy is any type of treatment that involves use of essential oils. These might be obtained from herbs, flowers or other plants.^19^ The compounds can be administered topically or through inhalation or water immersion.

#### 2.1 Aromatherapy for treatment of postoperative nausea and vomiting

This review^7^ aimed to assess the efficacy and safety of aromatherapy for treatment of postoperative nausea and vomiting. Sixteen controlled trials were included (n = 1,036 participants). Compared with placebo, aromatherapy reduced the use of rescue antiemetic medication (RR 0.60; 95% CI 0.37 to 0.97; seven RCTs; 609 participants; low quality of evidence). No difference between the groups was found regarding the following outcomes:

- severity of nausea, assessed on a visual analogue scale at the end of treatment (SMD -0.22; 95% CI -0.63 to 0.18; six RCTs; 241 participants; low quality of evidence); and
- proportion of participants without nausea at the end of treatment (RR 3.25; 95% CI 0.31 to 34.33; four RCTs; 193 participants; very low quality of evidence).

A specific analysis comparing peppermint aromatherapy versus placebo found no difference in severity of nausea at five minutes (SMD -0.18; 95% CI -0.86 to 0.49; four RCTs; 115 participants; low quality of evidence). No data were pooled for other outcomes.

Comparison of isopropyl alcohol aromatherapy with placebo showed that this treatment provided benefits regarding the following outcomes:

- time (in minutes) to 50% reduction of nausea score (SMD -1.10 minutes; 95% CI -1.43 to -0.78; 3 RCTs; 176 participants; moderate quality of evidence); and
- proportion of patients requiring antiemetics (RR 0.67; 95% CI 0.46 to 0.98; four RCTs; 215 participants; moderate quality of evidence).

No difference was found between the groups regarding patient satisfaction (RR 1.12; 95% CI 0.62 to 2.03; two RCTs; 172 participants; very low quality of evidence).

The overall methodological quality of the studies was considered low by the review authors. The adverse events were poorly reported, and no data were pooled. They considered that further studies would be imperative for solid conclusions to be drawn for practice. For further details and to access all analyses, refer to the original abstract, available from: http://cochranelibrary-wiley.com/doi/10.1002/14651858.CD007598.pub3/full.

#### 2.2 Aromatherapy for dementia

This review^8^ assessed the efficacy of aromatherapy for people with dementia. Seven RCTs were included (n = 428 participants). These compared the use of any fragrance from plants versus placebo aromatherapy. Overall, the studies included presented important uncertainties relating to methodological issues, low numbers of participants and lack of data reporting. The authors of this review retrieved individual participant data from one RCT that showed statistically significant differences favoring aromatherapy in the Cohen-Mansfield Agitation Inventory (CMAI) after four weeks of treatment (MD -11.1; 95% CI -19.9 to - 2.2; one RCT; 71 participants) and in behavioral symptoms according to the Neuropsychiatric Inventory (MD -15.8; 95% CI -24.4 to -7.2; one RCT; 71 participants). These results were conflicting with the results from another RCT, in which there was no difference according to the Neuropsychiatric Inventory scale (MD 2.80; 95% CI -5.84 to 11.44; one RCT; 63 participants). There was no difference in adverse events between aromatherapy and placebo (RR 0.97; 95% CI 0.15 to 6.46; two RCTs; 124 participants; very low quality of evidence). The authors did not pool any other results because of the high diversity of the clinical and methodological aspects of the RCTs. Better designed and better reported RCTs are still needed in order to reduce the uncertainties and to make practical recommendations. For further details and to access all analyses, refer to the original abstract, available from: http://cochranelibrary-wiley.com/doi/10.1002/14651858.CD003150.pub2/full.

#### 2.3 Massage plus aromatherapy for symptom relief in people with cancer

This review^9^ assessed the effects of massage with or without aromatherapy on relief of symptoms in people with cancer. Only two small RCTs (n = 117 participants) were included, and these provided isolated analyses on the effects of aromatherapy effects. Considering the sample size and methodological and reporting limitations of both RCTs, the authors were unable to pool any data regarding pain relief, psychological symptoms or quality of life. Therefore, no solid conclusions can be reached regarding the addition of aromatherapy to massage for symptom relief in cancer patients. For further details and to access all analyses, refer to the original abstract, available from: http://cochranelibrary-wiley.com/doi/10.1002/14651858.CD009873.pub3/full.

#### 2.4 Aromatherapy for pain management in labor

This review^10^ assessed the effects of aromatherapy for pain management in labor. Two RCTs (n = 535 participants) were included. The aim was to compare aromatherapy with another form of aromatherapy or with placebo, no treatment or other complementary interventions. Only one RCT (n = 513) compared aromatherapy with standard care, but no reliable data regarding pain intensity was reported. There were no differences in the risks of assisted vaginal delivery (RR 1.04; 95% CI 0.48 to 2.28; one RCT; 513 participants), caesarian section (RR 0.98; 95% CI 0.49 to 1.94; one RCT; 513 participants) or neonatal intensive care admission (RR 0.08; 95% CI 0.00 to 1.42; one RCT; 513 participants). The data from the other RCT (n = 22 participants) compared two forms of aromatherapy and will not be presented here. Until further results are available, no conclusion can be drawn regarding aromatherapy for pain management in labor. For further details and to access all analyses, refer to the original abstract, available from: http://cochranelibrary-wiley.com/doi/10.1002/14651858.CD009215/full.

### 3. Bioenergetics 

Bioenergetic analysis is a specific form of body psychotherapy. The bioenergetic approach has the aim of functioning through verbalization, corporal education and respiration techniques.^19^ Our search strategy did not retrieve any Cochrane systematic review addressing this intervention.

### 4. Chromotherapy

Chromotherapy or color therapy is a therapeutic technique that uses colors of the electromagnetic spectrum. The principle is that each color has an effect on the body and this may be converted into a therapeutic approach.^19^ Our search strategy did not retrieve any Cochrane systematic review addressing this intervention.

### 5. Family constellation

Family constellation is a psychotherapeutic method that aims to help patients by identifying hidden and transgenerational patterns of behavior in the family structure. It has the aim of leading towards resolution of conflicts within the family unit and within the individual perspective.^19^ Our search strategy did not retrieve any Cochrane systematic review addressing this intervention.

### 6. Flower therapy

Flower therapy is a therapeutic approach that uses flower-derived essences. There is a theory that the use of floral therapy might act on mental state and emotions.^19^ Our search strategy did not retrieve any Cochrane systematic review addressing this intervention.

### 7. Geotherapy

Geotherapy is defined as therapeutic use of a mixture of clay minerals and water in the form of cataplasm or mud baths applied to the skin.^19^ It is empirically used in esthetics and in treating dermatological and rheumatological diseases. Our search strategy did not retrieve any Cochrane systematic review addressing this intervention.

### 8. Hypnotherapy

The term hypnotherapy refers to a group of techniques that uses hypnosis to treat health-related conditions. It assumes that through concentration and relaxation maneuvers, the patient may be able to change undesired conditions and behaviors.^19^

#### 8.1 Hypnotherapy for treatment of irritable bowel syndrome

This review^11^ assessed the effects of hypnotherapy on the management of irritable bowel syndrome, in comparison with no treatment, waiting list or another therapeutic intervention. Four RCTs (n = 147 patients) were included. Because of the small sample size, poor reporting of outcomes and lack of methodological quality, no solid conclusions could be drawn. No meta-analysis was performed because there was important heterogeneity between the RCTs included. This systematic review was performed in 2010 and no assessment of the overall quality of the evidence was performed. The risk-of-bias assessment also needs to be updated to the new Cochrane standards. Until further RCTs and an updated SR have been conducted, the uncertainty regarding the use of hypnotherapy for treating irritable bowel syndrome remains. For further details and to access all analyses, refer to the original abstract, available from: http://cochranelibrary-wiley.com/doi/10.1002/14651858.CD005110.pub2.

#### 8.2 Hypnosis during pregnancy, childbirth and the postnatal period for preventing postnatal depression

This review^12^ had the aim of evaluating the benefits and harm of hypnosis for preventing postnatal depression, in comparison with the regular antenatal, natal and postnatal care. The authors of this review aimed to assess the development of postnatal depression, using a validated scale, and other secondary outcomes, such as postnatal psychosis, anxiety disorders, maternal mortality, suicidal ideation and death by suicide. One RCT was included (n = 63 participants). However, the data provided for evaluation of the effect of hypnosis was insufficient and poorly reported. Thus, further RCTs are imperative for solid conclusions regarding this topic to be reached. For further details and to access all analyses, refer to the original abstract, available from: http://cochranelibrary-wiley.com/doi/10.1002/14651858.CD009062.pub2/abstract.

#### 8.3 Hypnosis for induction of labor

This review^13^ aimed to evaluate the effects of hypnosis for induction of labor, compared with no intervention or any other interventions. The search was conducted in 2014 and no RCTs fulfilled the inclusion criteria. No conclusions can be drawn until appropriate RCTs have been developed. For further details, refer to the original abstract, available from: http://cochranelibrary-wiley.com/doi/10.1002/14651858.CD010852.pub2/abstract.

#### 8.4 Hypnosis for pain management during labor and childbirth

This review^14^ evaluated the effects of hypnosis for pain management in childbirth and labor. Seven RCTs and quasi-controlled trials (n = 1,213 participants) were included. They compared the use of hypnosis during or before labor versus placebo, no treatment or use of any analgesic drug or technique (control groups). The use of pharmacological pain relief or anesthesia was lower in the group that received self-hypnosis or hypnotherapy, compared with standard care (RR 0.73; 95% CI 0.57 to 0.94; eight RCTs; 2,916 participants; very low quality of evidence). No difference was found between the groups regarding satisfaction with pain relief (RR 1.06; 95% CI 0.94 to 1.20; one RCT; 264 participants; low quality of evidence) or spontaneous vaginal birth (RR 1.12; 95% CI 0.96 to 1.32; six RCTs; 2,631 participants; low quality of evidence). The overall quality of these data was downgraded because of design limitations, high inconsistency and imprecision. The studies included in the pooled analysis were very heterogeneous, and this needs to be considered in interpreting these results. No solid conclusion can be drawn until further studies have been conducted. The authors performed many analyses and subgroup investigations. For further details and to access all analyses, refer to the original abstract, available from: http://cochranelibrary-wiley.com/doi/10.1002/14651858.CD009356.pub3/full.

#### 8.5 Hypnosis for schizophrenia 

This review^15^ assessed the efficacy and safety of hypnosis for people with schizophrenia or schizophrenia-like illnesses, compared with any other treatment or standard therapy. Three RCTs were included (n = 149 participants). The main outcomes that the review aimed to investigate were: number of participants who dropped out before completion of the study; mental score, evaluated using the brief psychiatric rating scale (BPRS); movement disorders; and neurocognitive function.

Two RCTs evaluated hypnosis versus standard treatment. In both, none of the patients left the study early (within the first 12 weeks) in either group (risk difference [RD] 0.00; 95% CI -0.09 to 0.09; two RCTs; 70 participants). No difference was found between hypnosis and standard care regarding the BPRS scale (MD -3.63; 95% CI -12.05 to 4.79; one RCT; 60 participants). All other outcomes relating to hypnosis versus standard treatment were poorly reported. The authors also made head-to-head comparisons with music and relaxation techniques. Until further RCTs that are well designed and well reported have been conducted, no solid conclusions for practice can be drawn. For further details and to access all analyses, refer to the original abstract, available from: http://cochranelibrary-wiley.com/doi/10.1002/14651858.CD004160.pub3/full.

#### 8.6 Hypnotherapy for smoking cessation

This review^16^ evaluated the effects of hypnotherapy for smoking cessation, compared with no intervention and other intervention strategies. Eleven RCTs (n = 1,120 participants) that compared hypnotherapy with 18 different interventions were included.

Only one RCT (n = 20 participants) compared hypnotherapy with no treatment (a waiting list control), and this study found that there was a benefit for the hypnotherapy group regarding the probability of smoking cessation at 12 months (RR 19.00; 95% CI 1.18 to 305.88; one RCT; 20 participants). Compared with psychological treatments, hypnotherapy alone did not improve the probability of smoking cessation at six months (RR 0.93; 95% CI 0.47 to 1.82; two RCTs; 211 participants).

Despite the considerable number of RCTs included, they were highly heterogeneous regarding comparisons and methodological aspects, which prevented large quantitative synthesis. The risk of bias of each study also needed to be considered, and the overall quality of the evidence was not assessed in this SR. Until further RCTs have been conducted and this SR has been updated, no solid conclusions for practice can be reached. For further details and to access all analyses, refer to the original abstract, available from: http://cochranelibrary-wiley.com/doi/10.1002/14651858.CD001008.pub2/full.

### 9. Imposition of hands

Imposition of hands is, as defined in the Brazilian Ministry of Health’s Glossary for Integrative and Complementary Practices in Healthcare, “a secular therapeutic practice that implies a meditative effort to transfer vital energy (such as Qi or prana, i.e. a universal vital energy that permeates the cosmos and constitutes all that exists) through the hands, in order to re-establish the equilibrium of the human energy field, thereby assisting in the health-disease process.” It is believed that imposition of hands could be beneficial for decreasing the levels of pain, depression and anxiety.^19^ Our search strategy did not retrieve any Cochrane systematic review addressing this intervention.

### 10. Ozone therapy

Ozone is a molecule composed of three oxygen atoms. It has an unstable structure that makes it a powerful oxidant that can be administered in precise therapeutic doses. Some authors have claimed that it has health benefits under a variety of conditions characterized by hypoxic and ischemic syndromes.^20^

#### 10.1 Ozone therapy for treating foot ulcers in people with diabetes

This review^17^ assessed the efficacy and safety of ozone therapy for treating foot ulcers in patients with diabetes mellitus. Three RCTs (n = 212 participants) were included. One RCT (n = 101) compared the effects of ozone versus antibiotic treatment and showed that there was greater reduction in ulcer area in patients treated with ozone therapy (MD -20.54 cm^2^; 95% CI -20.61 to -20.47; one RCT; 101 participants), along with shorter duration of hospitalization (MD -8.00 days; 95% CI -14.17 to -1.83; one RCT; 101 participants); but that this did not alter the number of ulcers healed over 20 days (RR 1.10; 95% CI 0.87 to 1.40; one RCT; 101 participants). No adverse events were reported in either group. Another two RCTs (111 participants) compared the effects of ozone plus the usual care (debridement, daily wound dressings and moisturization) versus the usual care. There were no differences in the following: ulcer area (MD -2.11 cm^2^; 95% CI -5.29 to 1.07; two RCTs; 111 participants), number of ulcers healed (RR 1.69; 95% CI 0.90 to 3.17; two RCTs; 111 participants), amputation rate (RR 2.73; 95% CI 0.12 to 64.42; two RCTs; 111 participants) and adverse events (RR 2.27; 95% CI 0.48 to 10.79; two RCTs; 111 participants). Considering the small sample size and the methodological flaws of the studies included, the authors could not draw any solid conclusions for practice. For further details and to access all analyses, refer to the original abstract, available from: http://cochranelibrary-wiley.com/doi/10.1002/14651858.CD008474.pub2/full.

#### 10.2 Ozone therapy for treating dental caries

This review^18^ assessed the efficacy and safety of ozone therapy for controlling the progression of dental caries. Three RCTs were included (n = 137 participants). The authors of this SR aimed to evaluate the progression of caries in unrestored cases, use of further conventional treatment, time to intervention, cost, patient satisfaction and adverse events. All three RCTs included in this review evaluated local outcomes that the authors did not consider to be suitable for data pooling, so they did not do this. They concluded that there was no reliable evidence to support the use of ozone for treating dental caries. For further details and to access all analyses, refer to the original abstract, available from: http://cochranelibrary-wiley.com/doi/10.1002/14651858.CD004153.pub2/full.

## DISCUSSION

This overview of reviews included 16 systematic reviews (SRs) that assessed the use of 4 out of the 10 integrative practices that were recently added to the procedures available through the Brazilian public healthcare system (SUS). The specific topics found were apitherapy (four SRs), aromatherapy (four SRs), hypnotherapy (six SRs) and ozone therapy (two SRs). No Cochrane SRs was found regarding bioenergetics, family constellation, chromotherapy, geotherapy, flower therapy or hand imposition.

Overall, the Cochrane reviews included reported high-quality evidence regarding some outcomes from the use of apitherapy. All other evidence that was reported ranged in quality from unknown to moderate.

Honey dressings seemed to have some benefit over conventional dressings for the time needed to achieve partial healing of burn wounds, although this could be considered to be a surrogate outcome (if total healing were taken to be the clinically relevant outcome instead). Use of honey also seemed to reduce the frequency of coughing among children with acute coughs. Additionally, use of bee venom therapy seemed to prevent systematic allergic reactions to insect stings.

This overview made it clear that there are uncertainties regarding the efficacy and safety of the ten integrative practices that have recently been added to the procedures available through the Brazilian public healthcare system. Cochrane SRs are considered by many people to be the gold standard for evaluation of interventions within healthcare. Despite our wide-ranging search in the Cochrane database and broad inclusion criteria, we found that there was a lack of SRs investigating many topics (SRs were only available in relation to four out of these ten topics).

The absence of SRs relating to these topics may be indirectly indicative of the lack of controlled experimental studies assessing integrative practices. All of these practices need to be considered in the same way as any other intervention. There may have benefits, no effect or harm after their use. Any intervention, including integrative practices, is subject to adverse events and the safety component needs to be assessed in any evaluation.

This review has some limitations. Our search was conducted in a single database, even though the Cochrane Library is recognized as the most important database of systematic reviews. The limited data available on each topic is a consequence of the small number of studies, and the low quality of the evidence relates to the small sample sizes and risk of bias of the RCTs. Another point that should be noted is the huge variety of techniques for each integrative and complementary health practice that were considered in the primary studies that were included in these systematic reviews, which led to difficulty in identifying the overall effect of each intervention.

Regarding practical implications, except for a single case (apitherapy, i.e. specifically use of honey for partial healing of wound burns and for treating acute coughs; and bee venom therapy for preventing allergic reactions to insect stings), the use of these ten integrative practices that were recently incorporated into SUS does not seem to be supported by adequate evidence found in Cochrane SRs. Thus, for many integrative practices, no randomized clinical trial was found. This does not mean that no benefit exists, but it does mean that huge uncertainties remain, regarding the benefits and harm associated with use of such interventions.

Hence, it appears that the incorporation of these ten integrative practices into SUS is in disagreement with Brazilian Federal law number 12,401 (of April 2011), which establishes that health technologies, including medicines, orthoses, prostheses, diagnostic and therapeutic procedures and health care, can be incorporated into the public healthcare system (SUS) only when the National Commission for Incorporation of Technologies (CONITEC) finds scientific evidence of efficacy, accuracy, effectiveness and safety in relation to the drug, product or procedure under analysis that is accepted by the institution in charge of registration or authorization for use.^21^

Regarding the implications for research, this review found out that much remains to be done in relation to establishing what effects the IPs addressed here have for healthcare. RCTs with high methodological quality are recommended before any health intervention is brought into routine prescription and use. Subsequently, cost-effectiveness studies will need to be developed for the integrative practices that were proven to be effective and safe (as findings from RCTs). Economic studies analyzing these integrative practices in terms of their consequences for health and their economic burden are also worthwhile.

## CONCLUSION

This review identified 16 Cochrane systematic reviews that provided evidence, of a range of quality, in relation to ten new integrative practices that were recently incorporated into the Brazilian public healthcare system (SUS). Except for a few cases of apitherapy (honey dressings for partial healing of wound burns, honey to reduce coughing among children with acute coughs and bee venom to prevent allergic reactions to insect stings), none of the integrative practices addressed by the present review are supported by Cochrane SRs, because

- there is a lack of primary studies; or
- no Cochrane SRs exist (bioenergetics, chromotherapy, family constellation, flower therapy, geotherapy and imposition of hands); or
- the evidence found so far is insufficient for producing any sound conclusion. Evidence from additional sources regarding the effects of other integrative practices that have not been addressed by Cochrane SRs needs to be searched for.
